# Application of a Monopole Antenna Probe with an Optimized Flange Diameter for TDR Soil Moisture Measurement

**DOI:** 10.3390/s20082374

**Published:** 2020-04-22

**Authors:** Jacek Majcher, Marcin Kafarski, Andrzej Wilczek, Aleksandra Woszczyk, Agnieszka Szypłowska, Arkadiusz Lewandowski, Justyna Szerement, Wojciech Skierucha

**Affiliations:** 1 Department of Electrical Engineering and Electrotechnologies, Lublin University of Technology, Nadbystrzycka 38A, 20-618 Lublin, Poland; 2 Institute of Agrophysics, Polish Academy of Sciences, Doświadczalna 4, 20-290 Lublin, Poland; m.kafarski@ipan.lublin.pl (M.K.); a.wilczek@ipan.lublin.pl (A.W.); a.woszczyk@ipan.lublin.pl (A.W.); a.szyplowska@ipan.lublin.pl (A.S.); j.szerement@ipan.lublin.pl (J.S.); w.skierucha@ipan.lublin.pl (W.S.); 3 Institute of Electronic Systems, Warsaw University of Technology, Nowowiejska 15/19, 00-665 Warsaw, Poland; a.lewandowski@elka.pw.edu.pl

**Keywords:** antenna probe, dielectric permittivity, TDR, soil moisture, measurement techniques

## Abstract

Soil volumetric water content (*θ*) is a parameter describing one of the most important factors conditioning proper plant growth. Monitoring soil moisture is of particular importance in the rational use of water resources for irrigation, especially during periods of water scarcity. This paper presents a method of measuring soil moisture in the vicinity of the plant root system by means of a probe designed to be mounted on a mobile device used for precise plant irrigation. Due to the specific field conditions of the measurement, the design of the probe was proposed as a monopole antenna. Electromagnetic simulations of the probe were carried out with Ansys HFSS software to optimise its dimensions. Then a prototype of the probe was manufactured to conduct laboratory measurements with the use of a vector network analyser (VNA) working in the 20 kHz to 8 GHz frequency range. The VNA analyser was configured to work in the time-domain reflectometry (TDR) mode. From measurements of the time distance between reflections from the probe’s elements it is possible to calculate the bulk dielectric permittivity of the soil surrounding the probe. Next, based on commonly used soil moisture dielectric calibrations one can determine *θ* of the soil sample. The paper presents simulation results and laboratory tests of an antenna probe. Due to its tough and durable design, this type of probe gives the possibility of easy application in field conditions, which makes it especially suitable for mechanically demanding measurement systems. As the sensitivity zone is comparatively large, this probe is well-suited to measuring soil moisture in the vicinity of the plant root system.

## 1. Introduction

Water content in soil determines the biological and mechanical properties of soil [1] and has a decisive influence on plant development [2,3]. For plants to develop properly, constant access to water should be provided. Water demand strictly depends on the phase of plant development [4]. In order to correlate the above parameters, it is necessary to monitor plantations for water content in soil. The obtained results are necessary to decide on the moment of plant watering and the amount of water needed in this procedure.

The techniques used to measure soil moisture are divided into direct (oven-drying at 105 °C for 24 hours) and indirect (e.g. dielectric, thermal, neutron as well as measuring the matrix potential of water in soil). Currently, dynamic development of techniques based on the measurement of soil dielectric properties can be observed [5,6]. In this approach, soil moisture is determined based on the relative dielectric permittivity (*DP*) of soil. This parameter depends mainly on the high value of water’s *DP*, which at 20 °C is 80, while the *DP* of dry soil is 2–5 [7,8,9]. Therefore, the amount of water in soil has a significant impact on the *DP.*

The most common among the dielectric techniques are reflectometric ones, which are divided into the frequency domain reflectometry (FDR) and time domain reflectometry (TDR). They differ in the way of measuring *DP*. In the FDR technique, *DP* is determined for one (or more) frequencies of the applied electric field, usually by measuring the capacity or impedance of a sensor placed in the soil. Low frequencies are used here (<300 MHz, and most often not more than 175 MHz) [10], which results in a relatively low price of the device, but negatively affects the selectivity of moisture measurement in relation to electrical conductivity (*EC*), temperature, granulometric composition and other soil-dependent factors. 

The TDR soil moisture measurement technique assumes that the velocity of electromagnetic wave propagation in the soil depends selectively on its bulk dielectric permittivity (*DP*). This is true for most mineral soils with soil bulk *EC* values not bigger than about 400 mSm^-1^, representing the upper limit of soil salinity enabling the growth of most plants. For this purpose, a step or a needle impulse is generated at the system input, which moves along a coaxial cable of 50 Ω impedance to the probe, which forms a transmission line with soil as a dielectric. As a result of the impedance change at the point of connecting the coaxial cable to the probe and at the end of the probe the signal is reflected. Then the time delay between the characteristic points of the signal reflected from the end of the rod is analysed. On the basis of the obtained time, *DP* is calculated. This method is characterised by high measurement accuracy (1–2%) and has the advantage of using a generally accepted calibration proposed by Topp et al. [8,11,12,13,14]. Moreover, the TDR moisture measurement is not affected by the soil *EC* (salinity) of the tested medium, since a change in EC affects only the amplitude of the signal reflected from the end of the rod changes while the reflection time remains practically the same.

The assumptions in constructing the antenna-probe prototype were: (1) mechanically robust construction capable of withstanding multiple insertions into soils of various density, (2) no requirement for soil specific calibrations that are characteristic to low frequency FDR methods. For this reason, the TDR measurement technique described in the literature [15] was chosen. Moreover, the probe should be characterised by a large sensitivity zone so that soil moisture in the vicinity of the plant root system could be measured. Such solutions are also known from literature: e.g. a multi-rod probe described in [14,16,17] and a TDR array probe described in [18]. Due to the necessity to insert the probe into the soil several times in order to minimise the resistance to driving, the cross-sectional area of the probe should also be minimised, while ensuring high probe strength. The presence of stones or plant roots can cause problems in the correct installation of multi-rod probes, because individual rods may not be inserted into the soil in parallel and thus measurements may be subject to additional error or mechanical damage. To ensure better mechanical properties, the diameter of the bar should be increased. However, according to the authors of [19], thicker rods tend to compact the soil directly around the sensor, which may contribute to unrepresentative measurement loose soil conditions. For this reason, the above solutions are not suitable for field measurements with a mobile platform [20]. 

Such assumptions are met by a monopole antenna probe due to the use of only one rod. The literature is familiar with the studies on antenna probes. In paper [21] the authors present numerical FDTD simulations and laboratory tests concerning the monopole antenna. In the investigation presented in the paper [21], the authors used frequencies in the 50 MHz to 4 GHz range. Analyses of the dependence of the complex reflection coefficient on frequency showed the effect of the flange diameter on the amplitude of the first resonance frequency. Similar results are presented in papers [22,23] for silty soil. The telescopic antenna as a measuring probe was used by Qiwei et al. in the [24] work, where the resistance of the antenna was measured using VNA. These studies have found that the coefficient of determination of the calibration model was very low.

A similar solution is presented in the work [25]. The authors call their solution an open-ended probe with an antenna. It has been noticed that modification of the classical open-ended probe by adding a rod ensures a deeper measurement area. It is important in the case of precise irrigation of crops. In the works [21,22,23,24,25], the authors determined the frequency dependence of the real part of the dielectric permittivity. Then they determined relations between *θ* and the real part of dielectric permittivity. According to the [26] study, FDR analysis is more sensitive to temperature, salinity, bulk density and clay content than TDR. In the mentioned papers the signal is analysed in the frequency domain. Analysis of the signal in the time domain is difficult due to the length of the rod. Therefore, in the proposed probe, the rod has been extended to analyse the signal in the time domain. The above solution with an appropriate flange has been submitted for patent application [27]. 

The aim of this work is to develop a prototype monopole antenna probe for measuring soil moisture, to be mounted on a mobile device for precise watering of plants. The probe is to be characterised by the shortest possible time of soil moisture measurement, mechanical durability and high measurement accuracy. The motivation to carry out the research was to determine the usefulness of the TDR method for determination of moisture content in the antenna probe designs known from the literature. 

The scope of the work includes numerical simulation of the probe, model construction, laboratory measurements, optimisation of the flange diameter and determination of the calibration function (dependence of the electrical impulse travel time along the antenna rod as a function of *DP*).

## 2. Materials and Methods

The propagation of an electromagnetic wave in a porous non-magnetic material of negligible electric loss and dielectric dispersion conforms to the following dependence:

(1) $$DP≅\;{\left(\frac{c}{V}\right)}^{2},$$

where:

DP is the bulk dielectric permittivity,
*c* is the speed of light in a vacuum, and
*V* is the speed of the electromagnetic wave in a given material.

Since soil is a mixture of solid phase, air and water, its *DP* depends on the volumetric water content *θ*. Topp et al. [8] developed a universal calibration allowing one to calculate *θ* based on the TDR measured *DP*:

(2) $$DP=3.03+9.3\theta +146\theta^{2}-76.7\theta^{3}.$$

The above model is used for many types of mineral soils, as reported in [28]. Differences between the model and moisture determined by the drying method occur for soils of high porosity [28].

**Figure 1:**
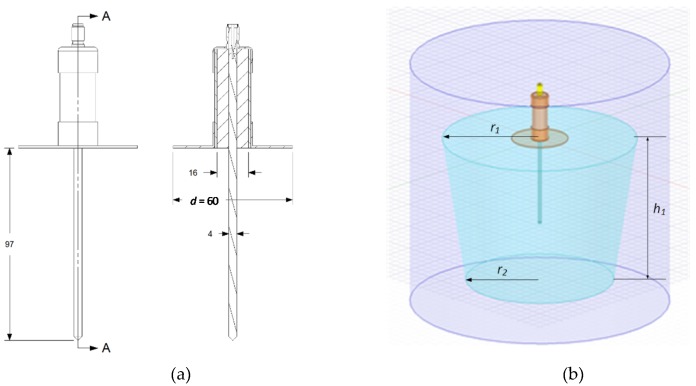
Probe view: (**a**) dimensions in mm, for the antenna probe with the flange of *d* = 60 mm in diameter, (**b**) 3D view of the probe model used in digital simulations, where: *r*_1_ is the upper radius of the container, *r*_2_ is the bottom radius of the container, and *h* is the height of the container.

The first stage of the research was to design the probe so that it met the assumed mechanical criteria. The next step was to simulate the probe’s performance in the Ansys HFSS software to optimize the probe performance. Then a physical model of the probe was made, consisting of a 50 ohm coaxial line terminated with an SMA-female connector (Figure 1a). The inside of the probe was filled with mineral filled cold-curing (2-component polyurethane cast resin ISO-PUR K 760). For the proportions used, the *DP* of the resin was 4.2 [29]. Due to the contact with soil and the possibility of corrosion, the probe rod was made of acid resistant steel. This type of steel ensures proper contact with soil that is constant over a long time regardless of the soil chemical composition, and that also has high mechanical strength. The dimensions of the probe are shown in Figure 1a, and a model of the probe for electromagnetic simulations is shown in Figure 1b. The shape of a soil container used for electromagnetic simulations was adjusted to the shape used later in laboratory measurements with the probe prototype.

**Figure 2:**
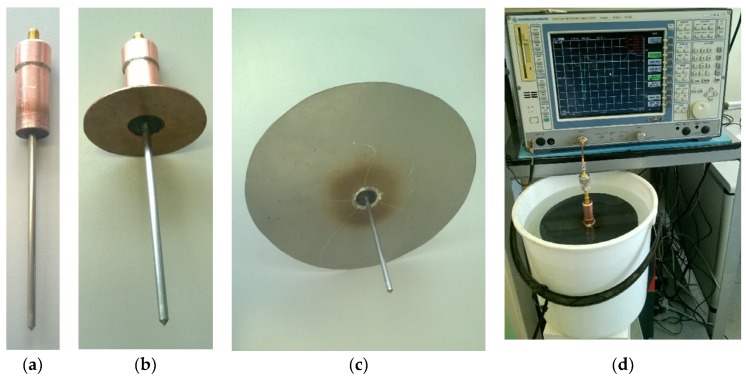
Actual probe model: (**a**) without flange, (**b**) with flange of diameter *d* = 60 mm, (**c**) with flange of diameter *d* = 200 mm and (**d**) test stand.

In order to verify the simulation results, the antenna probe prototype was built (Figure 2a). Two flanges with diameters of *d* = 60 mm (Figure 2b) and *d* = 200 mm (Figure 2c) were made for the probe. The experimental setup is shown in Figure 2d.

### 2.1. Digital Simulations

**Figure 3:**
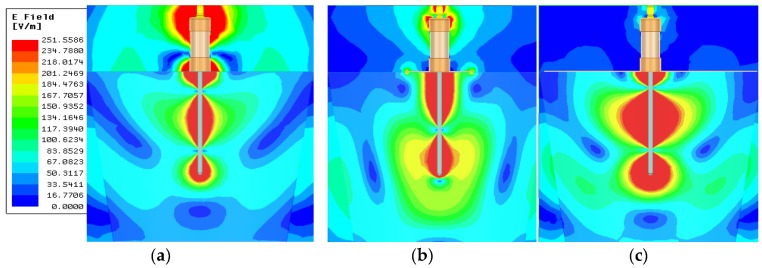
The electromagnetic field strength distribution in the material dielectric permittivity *(DP),* where *DP =* 2.44 for a probe with different flange diameters: (**a**) without flange, (**b**) *d* = 60 mm, and (**c**) *d* = 200 mm.

The container had a truncated cone shape with the radii of *r*_1_ = 210, *r*_2_ = 160 mm, and the height of *h*_1_ = 160 mm (Figure 1). Using Ansys HFSS software the electromagnetic field distribution was simulated for the probe with flange diameters of *d* = 40, 60, 80, 100, 120, 140, 160, 180, and 200 mm. The electromagnetic field distribution was performed at 1.55 GHz. Figure 3 shows selected electromagnetic field distributions.

### 2.2. VNA Measurements

In order to verify the simulation results, measurements on the antenna probe prototype were done in three media: air, distilled water and sand of variable water content (the granulation of sand was: 0.06–2.0 mm). Immediately before the VNA measurements, DP was measured with a commercial TDR soil moisture meter type FOM/mts equipped with a TDR field probe type FP/mts (https://www.e-test.eu). The antenna probe was then gently inserted into the soil to avoid any air gaps and to ensure a good contact with the soil and therefore to minimise the measurement errors. The measurements for each sample were taken ten times and average values were calculated.

The spectrum of the complex reflection coefficient was measured with a VNA (type ZVCE from Rohde & Schwarz) in the frequency range from 3.74 MHz to 3 GHz. The VNA was equipped with a TDR option and the measurement time window was set from 0 to 10 ns in 801 points in a linear time scale. The measurements were performed in laboratory conditions at a constant temperature of about 21 °C. Before the measurements were taken, the VNA was turned on for one hour to stabilize the temperature.

In order to be able to compare measurements to the digital simulations performed in Ansys HFSS software, the same parameters were introduced to the laboratory results as in the real model. Moisture content of sand samples changed from *θ* = 4.1% to saturation *θ* = 32% by adding distilled water. Each sand sample was thoroughly mixed with water and the surface was levelled to ensure a good flange contact. Next, the sample was sealed in an airtight vessel to avoid evaporation and placed in a temperature chamber WEISS WKL 100 at a constant temperature of 21 ± 0.5 °C for a period of 24 hours to obtain a uniform moisture distribution.

In order to verify the probe performance in media of various *EC*, sand samples were moistened with the distilled water and KCl solutions of *EC* from 20 to 566 mSm^−1^. To this end, several samples of dry soil were mixed with the same volume of KCl water solution. The values of *EC* for each solution differed to receive the same water content but different bulk electrical conductivity of the soil samples.

## 3. Results and Discussion

### 3.1. Influence of the Flange Dimension

When the probe has no flange, a significant part of the electromagnetic field is close to the probe housing and the SMA connector. This may result in a significant reduction in the amplitude of the impulse reflected from the end of the rod and thus hinder the correct interpretation of the reflectogram. In addition, the electromagnetic field extends well beyond the test material, leading to additional measurement errors. The use of a flange makes a larger part of the electromagnetic field stay between the flange and the rod, i.e. in the test material. Any further increase of the flange diameter results in better signal shielding than the probe body, but at the same time introduces a larger heterogeneity of the field around the probe rod (Figure 3c).

According to the digital simulations (Figure 3), adding a flange changes the distribution of the electromagnetic field around the probe rod. Without the flange the field lines partially close between the probe rod and its housing and cause current flow on the surface of the outer coaxial conductor. Adding a flange causes more of the signal reflected from the medium under test to reach the probe input instead of being scattered around its housing. On the other hand, to ensure proper contact with to the soil surface, especially in field conditions, the flange should be as small in diameter as possible. 

**Figure 4:**
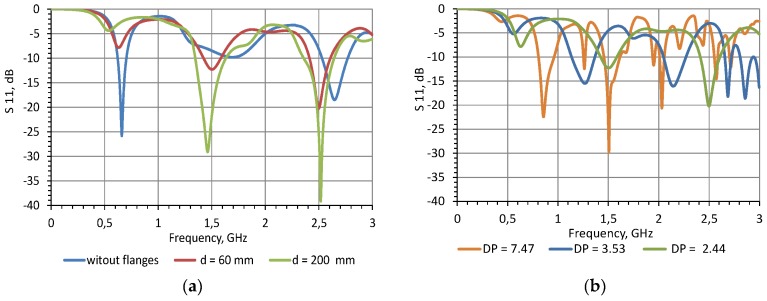
Amplitude of the reflection coefficient S11 versus frequency obtained from simulations in frequency domain (**a**) for different flange diameters (material *DP* = 2.44); and (**b**) for different *DP* (flange diameter d = 60 mm).

Since in many papers the measurements of the antenna probe are analysed in the frequency domain [21,22,23,24,25], the simulation in the frequency domain is shown in Figure 4.

The extraction of *DP* from the frequency-domain analysis of a monopole antenna is often complicated, as shown in [21,22]. Also, the TDR measurements can be performed with the use of much more energy-efficient devices than even the USB-powered portable one-port VNAs. Therefore, the present work focuses only on TDR analysis. 

### 3.2. Measurements inTime Domain

**Figure 5:**
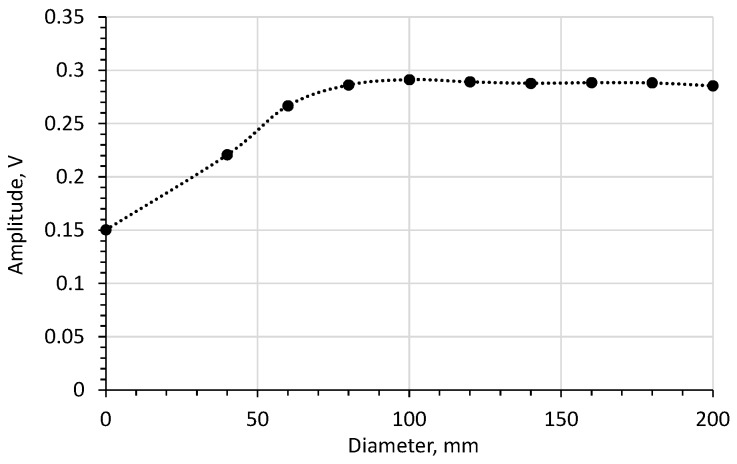
The effect of flange diameter on the amplitude of the impulse reflected from the end of the rod (material *DP* = 2.44).

In order to choose the optimum flange diameter, the influence of the flange size on the time-domain reflectogram was examined. Figure 5 shows the simulation results concerning the effect of flange diameter on the amplitude of the impulse reflected from the rod’s end.

Figure 5 shows that increasing the flange diameter above 70 mm does not increase the amplitude of the impulse reflected from the end of the rod. On the other hand, for a flange with a diameter of 60 mm, 95% of the maximum amplitude was obtained, which was regarded as acceptable. Therefore, a flange of 60 mm in diameter was chosen for further examination, as well as the 200 mm diameter flange, for reference. The study on the influence of flange diameter was also presented in the paper [21]. It was observed that the first resonant peak in frequency domain is the most prominent for flange diameter *d* = 150 mm and *DP* = 1.

**Figure 6:**
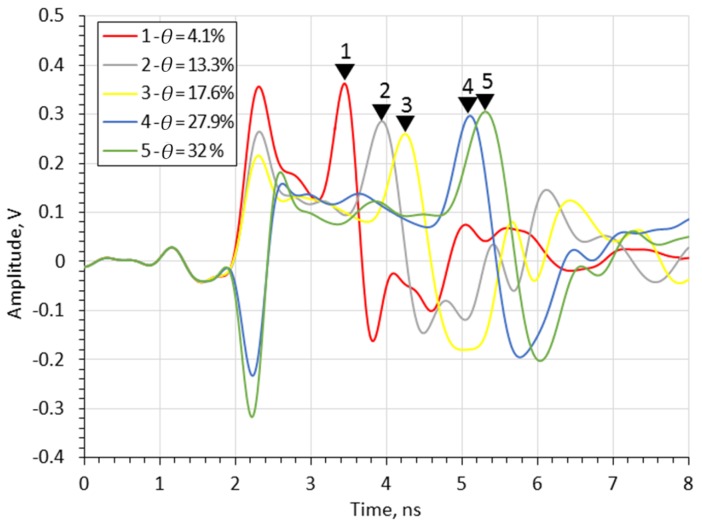
Time-domain waveforms measured by time-domain reflectometry (TDR) FOM/mts for sand with different *θ* values (given in the legend).

Since the probe is to be used for soil measurements, Figure 6 compares the results of the laboratory measurements of the probe with a 60 mm flange diameter, for different moisture contents of sand. The markers shown in the figure represent the time distance (in nanoseconds) from the beginning of the measurement time window to the impulse reflected from the end of the rod for each case. The number in marker labels increases with the sand moisture content *θ*.

**Figure 7:**
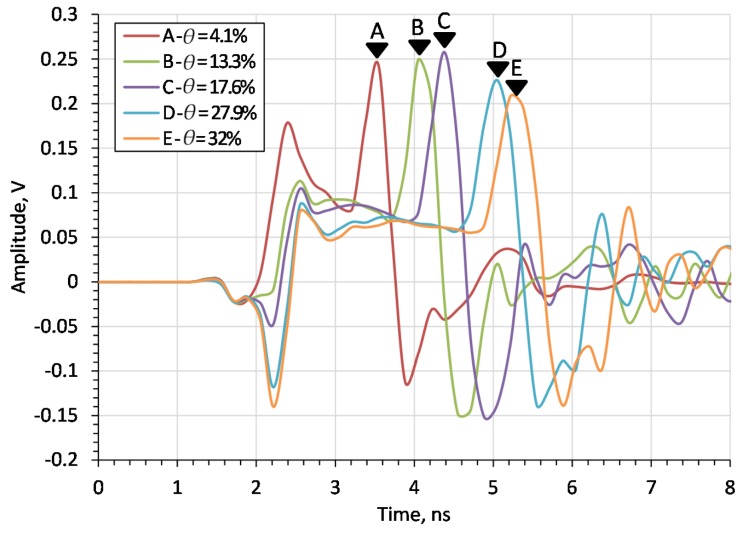
Time-domain digital simulations for sand with different *θ* values for flange diameters *d* = 60 mm.

Figure 6 shows that the time distance of the impulse reflected from the end of the rod increases with the *θ* of the medium, as expected. Figure 7 shows the results of the simulation in which the *DP* was equal to the *DP* of sand mixtures from Figure 6. The complex reflection coefficient resulting from the simulation was transformed into the time domain using the ADS (Keysight Advanced Design System) software. The frequency range of the simulation was 3.75 MHz to 3 GHz with a 3.75 MHz step. The markers shown in the figure represent time distance of the impulse reflected from the end of the rod for each run. As with simulation tests, the pulse travel time increases with the *θ*.

**Figure 8:**
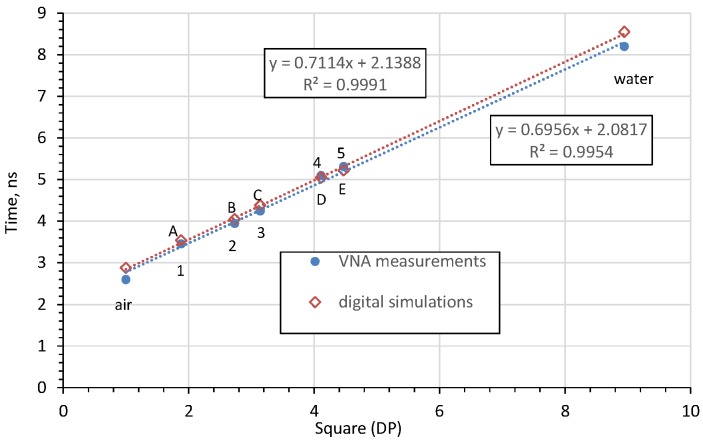
Comparison of the TDR-determined travel time of the impulse reflected from the end of the rod for the digital simulations and vector network analyser (VNA) measurements.

In order to check the correctness of the obtained results, measurements were additionally made in water (using the same container as used for soil) and in air in order to increase the measurement range. The experimental results were compared with the simulation results and are shown in Figure 8. For both laboratory tests and simulation, the relationship between the pulse travel time and the square root of *DP* is linear.

As the simulation and laboratory test results overlap, it is possible to formulate a calibration formula:

(3) $$\;DP={\left(1.4823\cdot T-3.344\right)}^{2},$$

where:

*T* is the TDR-determined travel time (ns) of the impulse reflected from the end of the rod (markers shown in the Figure 6 and Figure 7). The obtained calibration formula is similar to the one obtained in [14]. 

Having determined *DP* from the calibration Equation (3), the value of *θ* can be calculated from Equation (2). Since the values of the impulse reflection time from the end of the probe rod are linearly related to the square root of *DP*, as shown in Figure 8, it is possible to calibrate the device easily, using only two reference materials (e.g. water and air), as in the case of work [30].

**Figure 9:**
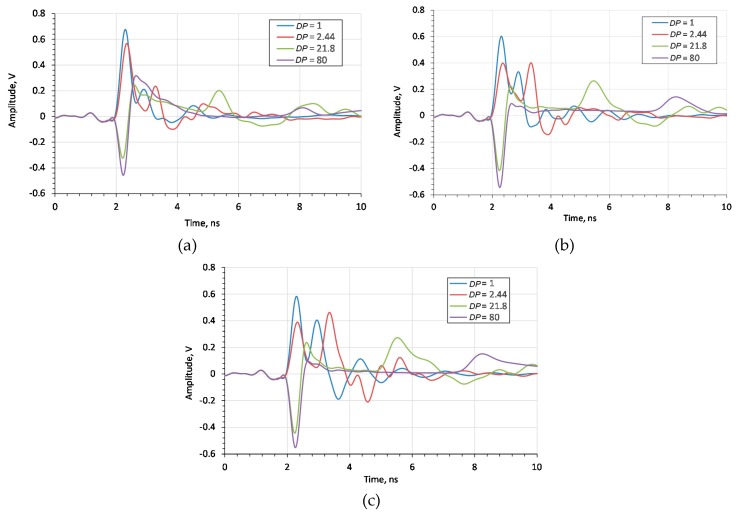
Reflectograms for various moisture contents obtained for the probe with different flange diameters: (**a**) without flanges, (**b**) *d* = 60 mm, (**c**) *d* = 200 mm.

In order to experimentally confirm that the 60 mm flange diameter is appropriate, measurements were taken for materials of different *DP* (air, dry sand, saturated sand and water). The measurement results are shown in Figure 9, as measured by the VNA.

It was determined that the diameter of the flange does not affect the travel time of the impulse reflected from the end of the rod but affects its amplitude. In the case of a probe without a flange, the time reading is difficult to identify due to the small amplitude of the signal reflected from the end of the probe rod for high *DP* materials. The amplitude differences for flanges with *d* = 60 mm and *d* = 200 mm are small.

**Figure 10:**
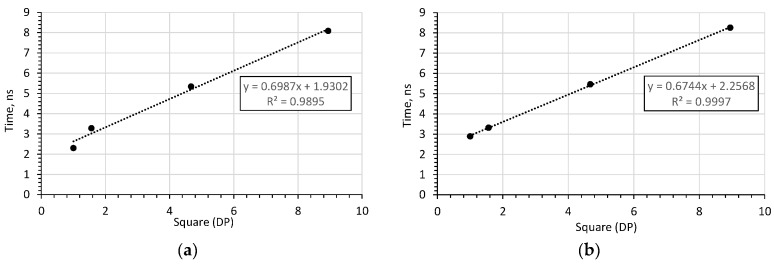

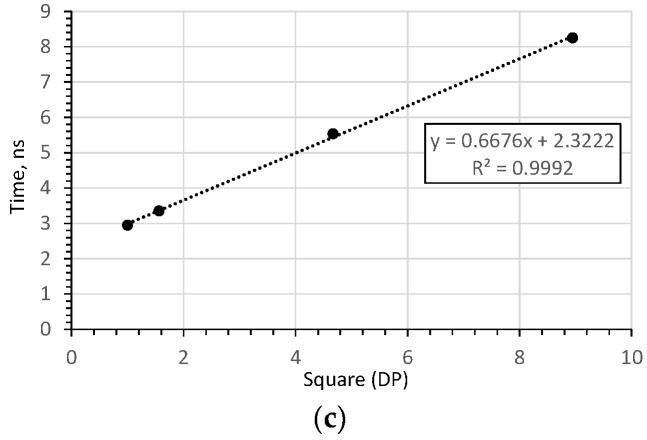
Trend lines fitted to the relation between the measured time of the impulse reflected from the end of the rod and the square root of *DP* of the respective measured media for the probe: (**a**) without flange, (**b**) *d* = 60 mm, (**c**) *d* = 200 mm.

Additionally, in order to assess the influence of the flange diameter, the mean-square value of relative errors of fitting a straight line to the measurements given in Figure 10 were calculated. The error values are listed in Table 1.

The relative error analysis shows that for a flange with a diameter of *d* = 60 mm, the TDR time characteristic of the *DP* is the most linear. This is particularly important when determining the calibration functions *θ* = *f* (*DP*) for the antenna probe. Similar conclusions were observed in study [25], where increasing the flange diameter above 2.5 times that of the probe rod length caused a decrease in the amplitude of the first resonance frequency signal.

### 3.3. Influence of Electrical Conductivity

**Figure 11:**
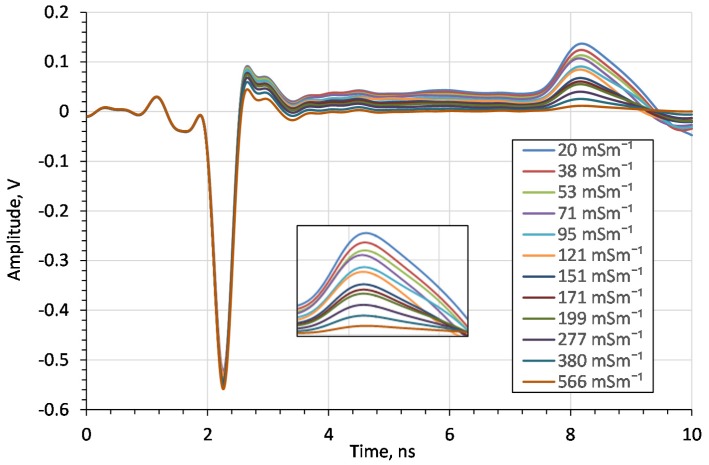
The impact of *EC* of water saline solutions on the time domain waveforms.

The amplitude of the impulse after passing through the lossy medium depends on the medium’s *EC*. For this purpose, the influence of the *EC* on TDR reflectograms, and thus on the moisture content measurement, was examined. Firstly, the reflectograms for saline water solutions of different electrical conductivity values are shown in Figure 11. 

From the above figure it can be seen that the bulk *EC* does not affect the impulse travel time but only affects its amplitude, like in the case of other TDR probes [31]. For *EC* above 380 mSm^−1^ the amplitude of the reflected impulse disappears. Therefore, the upper *EC* limit of the measured material is 380 mSm^−1^, which is above the bulk *EC* limit for most agricultural soils. The *EC* measuring range is similar to commercial probes. For example, for the probe WET-2 the *EC* measuring range is 0–300 mSm^−1^ [32].

**Figure 12:**
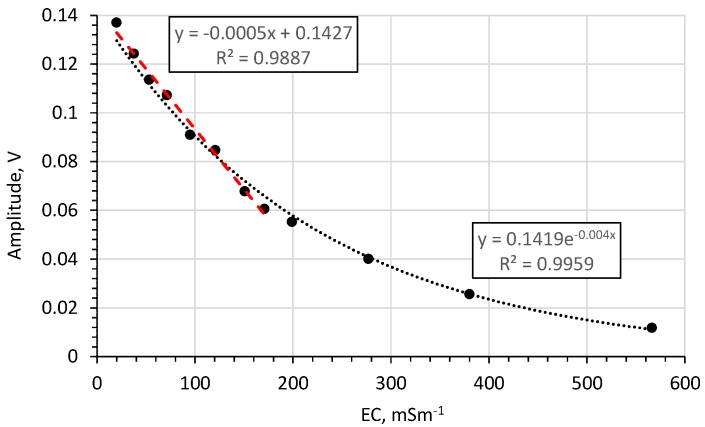
Effects of *EC* on the amplitude of the impulse reflected from the end of the rod for water solutions with different EC.

Figure 12 shows the amplitude of the impulse reflected from the end of the rod as a function of water solution with different *EC*s. This relationship can be fitted with an exponential function with a high coefficient of regression. Furthermore, for *EC*s in the range 20–171 mSm^−1^ this relationship is approximately linear.

**Figure 13:**
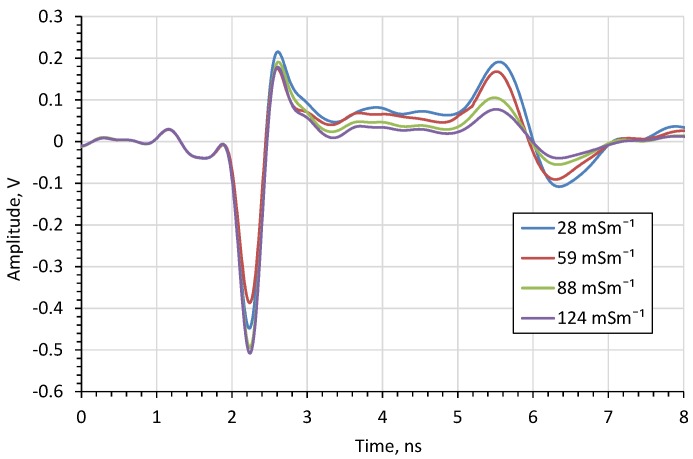
The impact of soil bulk *EC* (measured by TDR FOM/mts) on the time domain waveforms in sand.

Next, in order to verify the possibility of using the proposed antenna probe design in soils with different bulk *EC*s, measurements were also made in sand, for different bulk *EC*s but with the same *θ =* 34%. The results of the experiment are shown in Figure 13. From this figure, it can be concluded that the probe is suitable for measurements in soils with different bulk *EC*s. It was observed that similarly to water solutions of different salinity, the bulk *EC* of sand influences the amplitude of the signal reflected from the end of the probe.

**Figure 14:**
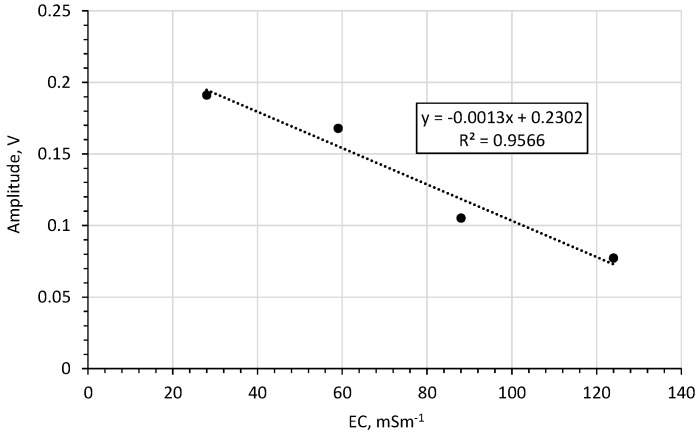
The impact of bulk *EC* on the amplitude of the impulse reflected from the end of the rod in sand.

Figure 14 shows the amplitude of the reflected impulse in the sand bulk *EC* function. 

For bulk *EC*s in the range 28–124 mSm^−1^ both for water (Figure 11) and for sand (Figure 14) this correlation is linear with a high coefficient of determination (0.99 for water and 0.96 for sand).

## 4. Conclusions

The paper presents simulation results and laboratory tests of an antenna probe. Due to its tough and durable design, this type of probe gives the possibility of easy application in field conditions, which makes it especially suitable for mechanically demanding measuring systems. As the sensitivity zone is comparatively big, this probe is well-suited to measuring soil moisture in the vicinity of the plant root system. The use of a flange increases the amplitude of the impulse reflected from the end of the rod, which is important for automatic procedures for analysing reflectograms. Additionally, it was noted that increasing the flange diameter above *d* = 70 mm does not significantly affect the amplitude of the impulse reflected from the end of the rod, with the highest linearity obtained for flanges with a diameter of *d* = 60 mm. Further increase of the flange diameter increases the non-linearity of the relations between the time of the impulse reflected from the end of the rod and the square root of *DP* of the measured soil material. 

Since the dependence of the reflected impulse travel-time on the square root of *DP*, both for laboratory and digital simulation tests, and since the relationship is approximately linear (with the determination coefficient above 0.99), it is possible to calibrate the probe using two reference materials (e.g. air and water).

The reflected impulse travel-time does not depend on the bulk *EC*. Moreover, for bulk *EC*s in the range 28–124 mSm^−1^, both for water and for sand, this correlation is linear. This means that the probe can be used to determine the bulk *EC* from the amplitude of the reflected impulse from the end of the rod, especially in the “linear” range.

Further research will involve construction of the field prototype of the probe and testing the performance of probe mounted on the mobile device in real field conditions.

## Figures and Tables

**Table 1:** Relative errors for different flange diameters.

Relative Errors			
Square Root of (*DP*)	Without Flanges	*d* = 60 mm	*d* = 200 mm
1	12.41%	1.29%	1.39%
1.56	8.71%	0.26%	0.35%
4.67	2.93%	1.03%	1.79%
8.94	1.08%	0.31%	0.53%
